# Dentists’ Relation Not Confidential

**Published:** 1895-09

**Authors:** 


### Dentists' Relations Not Confidential.

During the trial of the case of The People vs. Stonewall J. De France for forgery, the defense attempted to raise a question of identity, for which purpose they showed that the teeth of the accused were entirely different from those of the person committing the forgery as alleged. In rebuttal, a dentist of Detroit was cited who testified that subsequent to the date of the forgery he had inserted three false teeth in the place of two incisors for De France. The case was carried up on appeal to the Supreme Court of Michigan, on the ground that the trial court had erred in admitting the dentist's testimony, claiming that his knowledge was privileged as between physicians or surgeons and patients. The Supreme Court affirmed the verdict of conviction and held that the terms "dentist" and "physician or surgeon," as the latter are used in the statute covering this point, are not interchangeable and that a dentist's relations with his patient can not be considered confidential as is the case with a physician or surgeon.

